# Contrasting Roles of Intraspecific Trait Variability and Species Turnover in Shaping Functional Composition During Vegetation Restoration on the Loess Plateau

**DOI:** 10.1002/ece3.71808

**Published:** 2025-07-13

**Authors:** Yuting Yang, Zhifei Chen, Xiangtao Wang, Junqin Li, Yang Gao, Puchang Wang, Zhongming Wen

**Affiliations:** ^1^ School of Life Sciences Guizhou Normal University Guiyang Guizhou China; ^2^ College of Life Sciences Guizhou University Guiyang Guizhou China; ^3^ School of Geography and Environmental Sciences/Karst Research Institute Guizhou Normal University Guiyang Guizhou China; ^4^ College of Grassland Agriculture Northwest A&F University Yangling Shaanxi China

**Keywords:** community ecology, community functional composition, functional traits, intraspecific trait variability, loess plateau

## Abstract

Understanding how ecological communities respond to environmental variation is one of the fundamental goals in ecology. Both intraspecific trait variability (ITV) and species turnover contribute to shaping community functional composition, yet their relative importance in mediating community responses remains insufficiently resolved. We investigated the roles of ITV and species turnover in influencing community functional composition under natural and *Robinia pseudoacacia* plantation restoration on the Loess Plateau. We examined functional traits associated with photosynthetic performance, water‐use strategies, and nutrient acquisition to elucidate the adaptive mechanisms of plants. Overall, ITV accounted for 38% and 43% of the community functional composition of natural vegetation and *R. pseudoacacia* plantation on average, respectively. ITV accounted for a substantial proportion of the variation in community functional composition. Notably, positive covariation between the contributions of species turnover and ITV was observed for leaf traits in response to environmental variation. *Robinia pseudoacacia* plantation exhibited greater ITV (33%–53%) compared to natural vegetation, whereas environmental factors exerted stronger explanatory power on the community composition of natural vegetation. These results underscore the adaptive significance of key functional traits in driving intraspecific adaptations and provide insights into strategies for ecological restoration on the Loess Plateau. Moreover, our study emphasizes the critical role of ITV in predicting community responses to environmental variability, offering implications for trait‐based ecological research and the sustainable management of restored ecosystems.

## Graphical abstract

Understanding how ecological communities respond to environmental variation is a fundamental goal in ecology. Both intraspecific trait variability (ITV) and species turnover contribute to shaping community functional composition, yet their relative importance in mediating community responses remains insufficiently resolved. Our study emphasizes the critical role of ITV in predicting community responses to environmental variability, offering implications for trait‐based ecological research and the sustainable management of restored ecosystems.

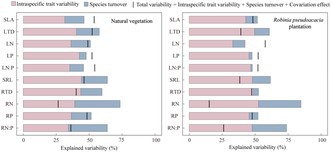

## 1 Introduction

Functional traits—encompassing morphological, physiological, and phenological characteristics—serve as critical indicators of resource use and fitness. These functional traits are instrumental in linking environmental conditions to community functional structure and ecosystem functioning (Funk et al. 2017; Yan et al. 2023). A trait‐based framework offers restoration ecology a robust scaffold on which to apply fundamental ecological theory to maintain resilient and functioning ecosystems in a rapidly changing world (Laughlin 2014). Using trait‐based methods, ecologists would have greatly advanced in understanding community functional responses to environmental changes (Díaz 2025; Liu et al. 2024). Community functional composition is the key index for quantitative analysis of functional traits at the community level, which can directly affect ecosystem processes and functions (Niu et al. 2016). Environmental variations influence community functional composition by driving changes in intraspecific trait variability (ITV, reflecting phenotypic plasticity and genetic differentiation within species) and species turnover (involves changes in species relative abundance and occurrence) (Lepš et al. 2011; Sanders et al. 2025). The relative contribution of ITV and species turnover to the changes in community functional composition in response to environmental variations has implications for the resistance of plant communities to those variations (Hurtado et al. 2020; Violle et al. 2012). A greater role of ITV suggests enhanced adaptive capacity within species, thereby increasing community resistance. Conversely, a stronger influence of species turnover indicates reduced community resistance to environmental changes (Lü et al. 2018).

Historically, trait‐based ecology has predominantly focused on interspecific variation, often assuming that within‐species trait variability is negligible compared to differences among species (Araújo et al. 2011; Wang et al. 2022). This perspective treats species as functionally static entities, disregarding the potential for individuals to adjust their traits in response to environmental heterogeneity (Puglielli et al. 2024). However, emerging evidence suggests that ITV contributes significantly to community‐level trait variation, influencing ecological strategies and enhancing community stability under changing environmental conditions (Lang et al. 2020; Rixen et al. 2022). Disentangling the relative contributions of ITV and species turnover is therefore pivotal for advancing our understanding of community assembly processes and predicting ecological responses to global change (Fenollosa et al. 2024; Violle et al. 2012).

The Loess Plateau in China provides a compelling system for investigating the roles of ITV and species turnover in shaping community functional structure. This region has undergone extensive ecological restoration efforts to mitigate severe soil erosion and ecosystem degradation (Fu et al. 2023; Jian et al. 2015). While afforestation initiatives have significantly increased vegetation cover and improved soil conditions, they have also raised concerns about long‐term sustainability, particularly regarding soil desiccation and water availability (Feng et al. 2016; Huang and Shao 2019). To date, research in this region has primarily focused on the impacts of these interventions on vegetation productivity and soil moisture dynamics (Jia et al. 2024; Su et al. 2021; Yang et al. 2022). In contrast, the role of ITV in mediating plant functional strategies remains underexplored.

In this study, we quantified the relative contributions of ITV and species turnover to community functional composition in natural vegetation and *Robinia pseudoacacia* plantation communities on the Loess Plateau. We focus on both aboveground and belowground traits that are central to plant resource acquisition and growth strategies, such as leaf and root traits associated with resource use and fitness. Specifically, we address the following questions: How does ITV vary among different functional traits? How do the relative contributions of ITV and species turnover differ between natural and *R. pseudoacacia* plantation communities? To what extent do ITV and species turnover mediate community functional responses to environmental variations in this fragile and changing environment? By addressing these questions, our study offers new insights into the mechanisms underlying vegetation adaptation and resilience in a critical ecological restoration region.

## 2 Materials and Methods

### 2.1 Study Area and Vegetation Investigation

**FIGURE 1:**
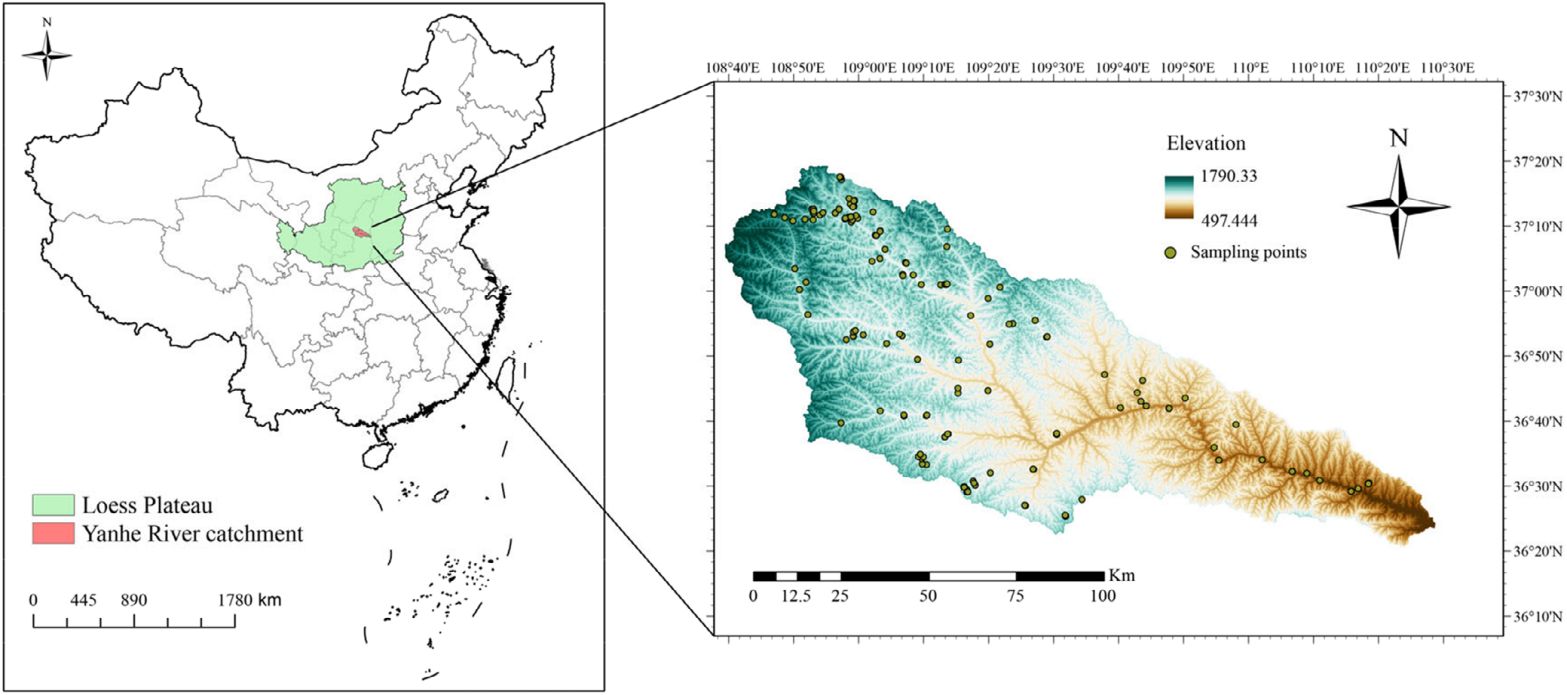
Location of the Yanhe River catchment and distribution of sampling points.

The study site is located within the Yanhe River catchment, located in the central region of China's Loess Plateau (108°4′–110°28′ E, 36°23′–37°17′ N; Figure 1), encompassing a total area of 7687 km^2^, approximately 90% of which is hilly. The catchment spans a length of 286.9 km and experiences a semi‐arid climate characterized by heavy seasonal rainfall, periodic flooding, and subsequent droughts. The mean annual precipitation (MAP) is 505 mm, with a range of 431–539 mm. The mean annual temperature (MAT) is 9°C, with a range of 8°C–13°C. Within the Yanhe River catchment, the MAP and MAT show a gradient decreasing trend from southeast to northwest (Figure S1). The distribution of vegetation changes obviously along the environmental gradient. Natural vegetation in the south of the Yanhe River catchment is dominated by *Quercus mongolica* and *Pinus tabuliformis Carrière*. *Caragana intermedia*, *Bothriochloa ischaemum* , and *Sophora davidii* are the main natural vegetation in the middle of the Yanhe River catchment. Natural vegetation in the north of the Yanhe River catchment is dominated by *Artemisia stechmanniana*, *Stipa bungeana*, *Lespedeza davurica*, and *Stipa grandis* . *Robinia pseudoacacia* is extensively planted within the catchment.

Field investigations were conducted from mid‐August 2017 to 2021 during the peak community biomass period. Sampling points were selected to include both natural and *R. pseudoacacia* plantation under comparable site conditions. Natural vegetation was represented by stable plant communities, while *R. pseudoacacia* plantation samples consisted of *R. pseudoacacia* forests. Vegetation surveys were performed using quadrats of different sizes: 10 × 10 m for tree quadrats, 5 × 5 m for shrub quadrats, and 1 × 1 m for herb quadrats.

Environmental factors that have an important impact on vegetation distribution were selected, including MAT, MAP, and Aspect. MAT and MAP were interpolated using ANUSPLIN software based on meteorological data from the Yanhe River catchment and surrounding stations. Aspect was extracted from a digital elevation model of the region.

### 2.2 Trait Measurements

Dominant species—those contributing more than 80% of the relative importance value in each quadrat—were selected for plant functional trait measurements (Table S1). We focused on five leaf traits (specific leaf area [SLA], leaf tissue density [LTD], leaf nitrogen content [LN], leaf phosphorus content [LP], and the leaf N:P ratio [LN:P]) and five root traits (specific root length [SRL], root tissue density [RTD], root nitrogen content [RN], root phosphorus content [RP], and the root N:P ratio [RN:P]). These traits are associated with nutrient uptake and growth strategies.

In each quadrat, 10 healthy, unshaded leaves from each dominant species were sampled to measure leaf traits, and 10 fine roots (diameter < 2 mm) were collected from the same individuals to measure root traits. Leaf and root samples were scanned using a portable scanner (CanoScan LiDE 120). Leaf area was determined using ImageJ software, and root length was measured using WINRHIZO software (Regent Instruments Inc., Quebec City, Canada). Samples were oven‐dried to a constant mass before weighing. SLA was calculated as leaf area divided by leaf dry mass, and LTD was calculated as leaf dry mass divided by leaf volume. SRL was determined as root length divided by root dry mass, and RTD as root dry mass divided by root volume. LN and RN were measured using an auto‐Kjeldahl instrument (Kjektec System 2300 Distilling Unit, Foss, Sweden), while LP and RP were determined using the molybdenum‐antimony colorimetric method.

### 2.3 Statistical Analysis

#### 2.3.1 Community‐Weighted Means

Community‐weighted mean (CWM) trait values, commonly used to characterize community functional composition, were calculated for each plot by weighting trait values by the relative abundance of each species:

$$\mathrm{CWM}=\sum_{i=1}^{n}p_{i}\times {\text{trait}}_{i}$$

where, *p*_i_ is the relative importance value of species *i* in the community, trait_i_ is the mean trait value of species *i* in each plot.

#### 2.3.2 Relative Contribution of Species Turnover and ITV

The calculation method of species turnover and ITV refer to Lepš et al. (2011). To disentangle the relative contributions of ITV and species turnover to community functional composition, we used the sum of squares (SS) decomposition method proposed by Lepš et al. (2011). For each plot‐level CWM trait, we calculated three types of SS: (1) total CWM traits (SS_*specific*_), using relative importance of species in a given plot and trait values measured in this plot, including both ITV and species turnover effects; (2) fixed CWM traits (SS_*fixed*_), using relative importance of species in a plot and trait values averaged across all plots, including only the effects of species turnover; (3) intraspecific CWM traits (SS_*intra*_), which is the differences between “specific” and “fixed” average. The covariation (SS_*cov*_) between ITV and species turnover was calculated as follows: SS_*cov*_ = SS_*specific*_—SS_*fixed*_—SS_*intra*_.

All statistical analyses were performed using R software (R 4.2.1; R Development Core Team). The individual and common effects of MAP, MAT, and aspect on ITV and species turnover were estimated using the variation decomposition analysis (“rdacca. hp” package).

## 3 Results

### 3.1 Relative Contributions of ITV and Species Turnover

**FIGURE 2:**
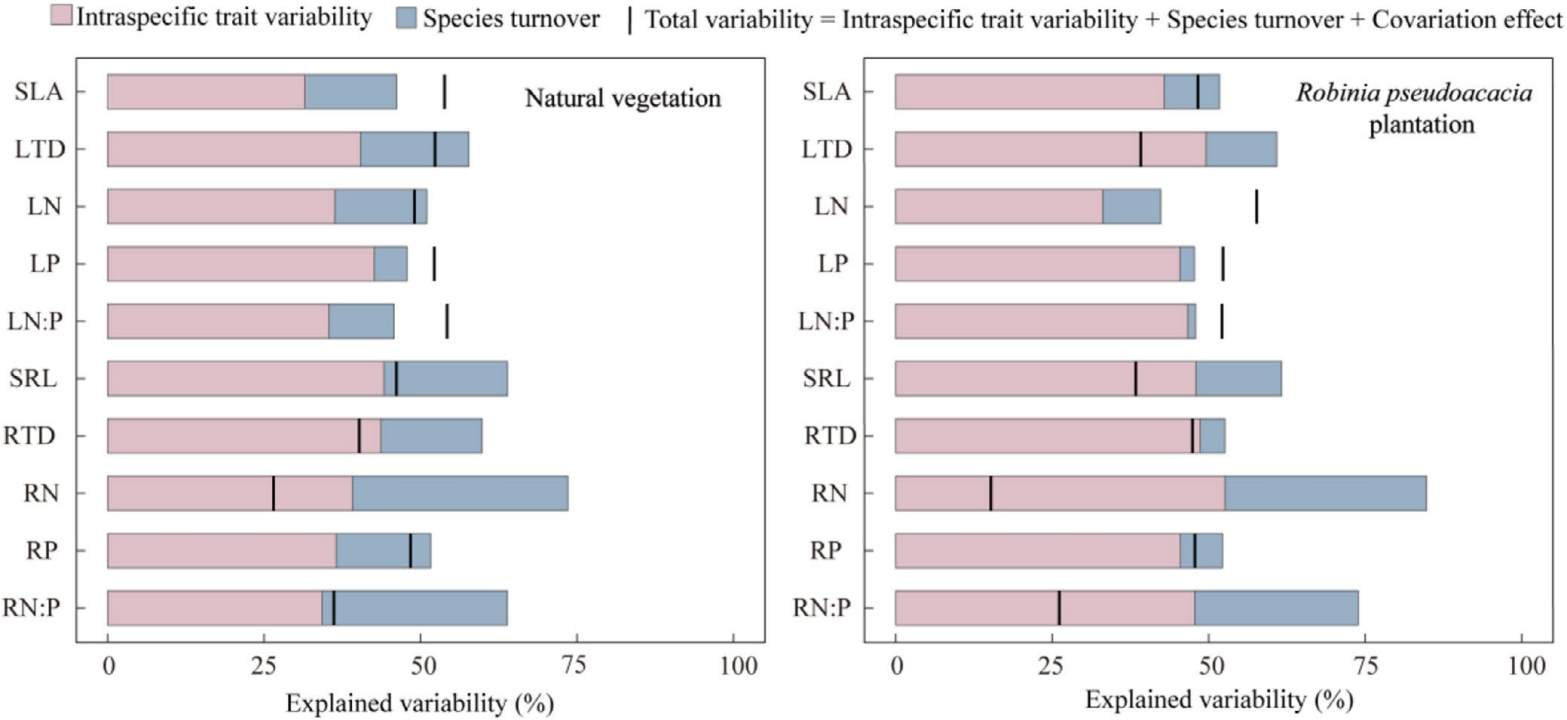
Results from sum of squares decomposition analyses showing the relative importance of intraspecific trait variability versus species turnover in explaining the community‐weighted mean trait values of natural and *Robinia pseudoacacia* plantation. Black bars represent total variability. The space between the top of the column and the bar indicates the effect of covariation; positive covariation occurs when the bar is above the column, and negative covariation occurs when it crosses the column. Traits: SLA, specific leaf area; LTD, leaf tissue density; LN, leaf nitrogen content; LP, leaf phosphorus content; LN:P, leaf N:P ratio; SRL, specific root length; RTD, root tissue density; RN, root nitrogen content; RP, root phosphorus content; RN:P, root N:P ratio.

The SLA, LN, and RP of natural vegetation were lower than those of *R. pseudoacacia* plantation. Natural vegetation exhibited a wider range in most functional traits, whereas *R. pseudoacacia* plantations demonstrated relatively concentrated smaller ranges (Table S2). ITV emerged as the primary driver of variation in both leaf and root traits (Figure 2). For natural vegetation communities, ITV explained 31%–44% of the total variation in community‐level functional traits, whereas in *R. pseudoacacia* plantation, ITV accounted for an even greater proportion, ranging from 33% to 53%. In contrast, species turnover contributed relatively little, with an average contribution of 12%. Overall, *R. pseudoacacia* plantation demonstrated a stronger influence of ITV on functional composition compared to natural vegetation. Among the functional traits, ITV was particularly high for LN (36%), LP (43%), and LN:P (35%), but was lower for specific leaf area (SLA, 31%).

Covariation analyses revealed distinct patterns. In natural vegetation, positive covariation between ITV and species turnover was observed for SLA, LP, and LN:P, while root traits exhibited negative covariation. Conversely, in *R. pseudoacacia* plantation, negative covariation predominated for most traits, with the exception of LN, LP, and LN:P, which showed positive covariation (Figure 2).

### 3.2 ITV and Species Turnover in Response to Environmental Factors

**FIGURE 3:**
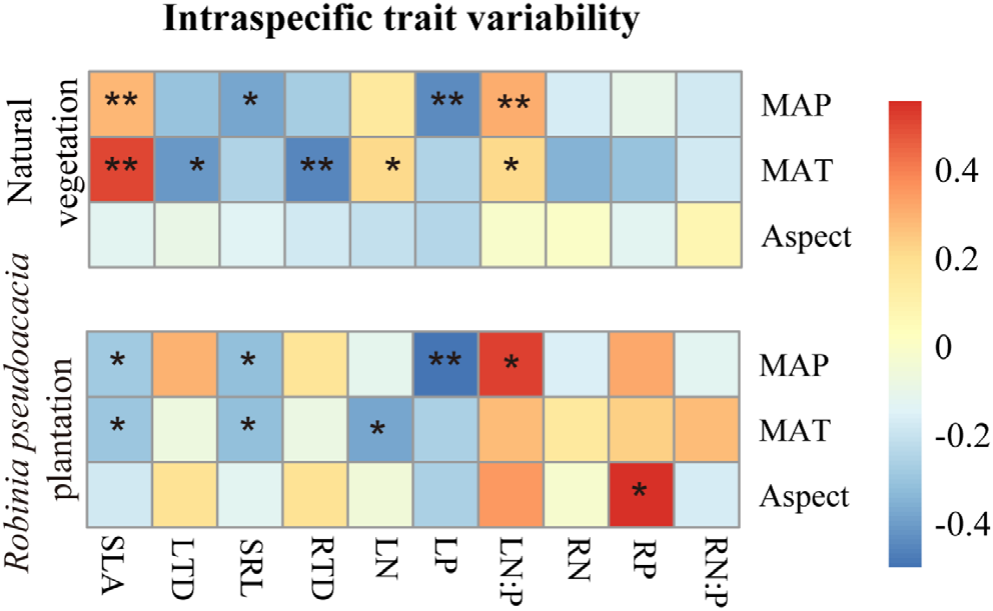
Relationship heat map between intraspecific trait variability in natural vegetation and *Robinia pseudoacacia* plantation and mean annual precipitation (MAP), mean annual temperature (MAT), and Aspect SLA, specific leaf area; LTD, leaf tissue density; LN, leaf nitrogen content; LP, leaf phosphorus content; LN:P, leaf N:P ratio. SRL, specific root length; RTD, root tissue density; RN, root nitrogen content; RP, root phosphorus content; RN:P, root N:P ratio. * and ** indicate statistical significance at *p* < 0.05 and *p* < 0.01, respectively.

The SLA, LN, and LN:P of natural vegetation showed significant positive relationships with MAP and MAT (*p* < 0.05); the SLA, RN, and RN:P of *R. pseudoacacia* plantation showed significant positive relationships with MAT (*p* < 0.05; Figure S2). In natural vegetation, ITV of SLA and LN:P showed significant positive relationships with MAP and MAT (*p* < 0.05), while ITV of LTD and RTD showed negative significant relationships with MAT; ITV of SRL and LP showed negative significant relationships with MAP (*p* < 0.05; Figure 3). In *R. pseudoacacia* plantation, ITV of SLA and SRL showed negative significant relationships with MAP and MAT (*p* < 0.05); ITV of LN showed negative significant relationship with MAT (*p* < 0.05), and ITV of LN:P showed significant positive relationships with MAP (*p* < 0.05; Figure 3).

**FIGURE 4:**
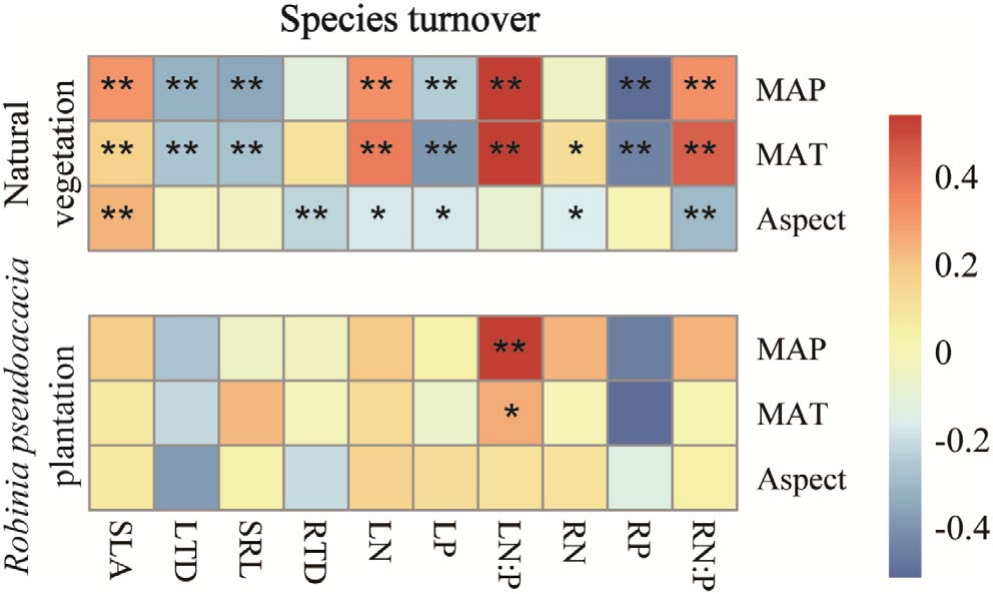
Relationship heat map between species turnover in natural vegetation and *Robinia pseudoacacia* plantation and mean annual precipitation (MAP), mean annual temperature (MAT), and Aspect. SLA, specific leaf area; LTD, leaf tissue density; LN, leaf nitrogen content; LP, leaf phosphorus content; LN:P, leaf N:P ratio. SRL, specific root length; RTD, root tissue density; RN, root nitrogen content; RP, root phosphorus content; RN:P, root N:P ratio. * and ** indicate statistical significance at *p* < 0.05 and *p* < 0.01, respectively.

Species turnover also varied in response to environmental variations. In natural vegetation, species turnover of SLA, LN, LN:P, and RN:P showed a significant positive relationship with MAP and MAT (*p* < 0.05), while species turnover of LTD, SRL, LP, and RP exhibited a negative significant relationship with MAP and MAT (*p* < 0.05). Species turnover of LN, LP, RN, and RN:P showed a negative significant relationship with aspect (*p* < 0.05). In *R. pseudoacacia* plantation, species turnover of LN:P showed significant positive relationships with MAP and MAT (*p* < 0.05), whereas species turnover of RP displayed a negative significant relationship with MAT (*p* < 0.05; Figure 4).

### 3.3 Effects of Environmental Factors on ITV and Species Turnover

**FIGURE 5:**
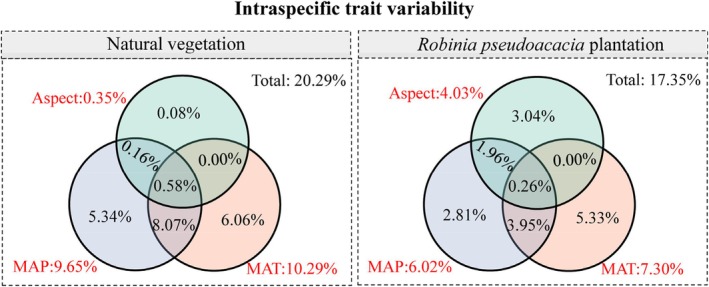
Venn diagram of variation partitioning analysis (VPA), illustrating the individual and common effects of mean annual precipitation (MAP), mean annual temperature (MAT), and aspect on intraspecific trait variability in natural vegetation and *Robinia pseudoacacia* plantation.

MAP, MAT, and aspect jointly explained a greater fraction of ITV variation in natural vegetation and *R. pseudoacacia* plantation, which were 20.29% and 17.35%, respectively, based on variation partitioning analysis. In natural vegetation, MAT and MAP contributed 10.29% and 9.65%, respectively, to the ITV, with aspect contributing minimally (0.35%) to the ITV. In *R. pseudoacacia* plantation, the individual effects of MAT, MAP, and aspect on the ITV were 7.30%, 6.02%, and 4.03%, respectively (Figure 5).

**FIGURE 6:**
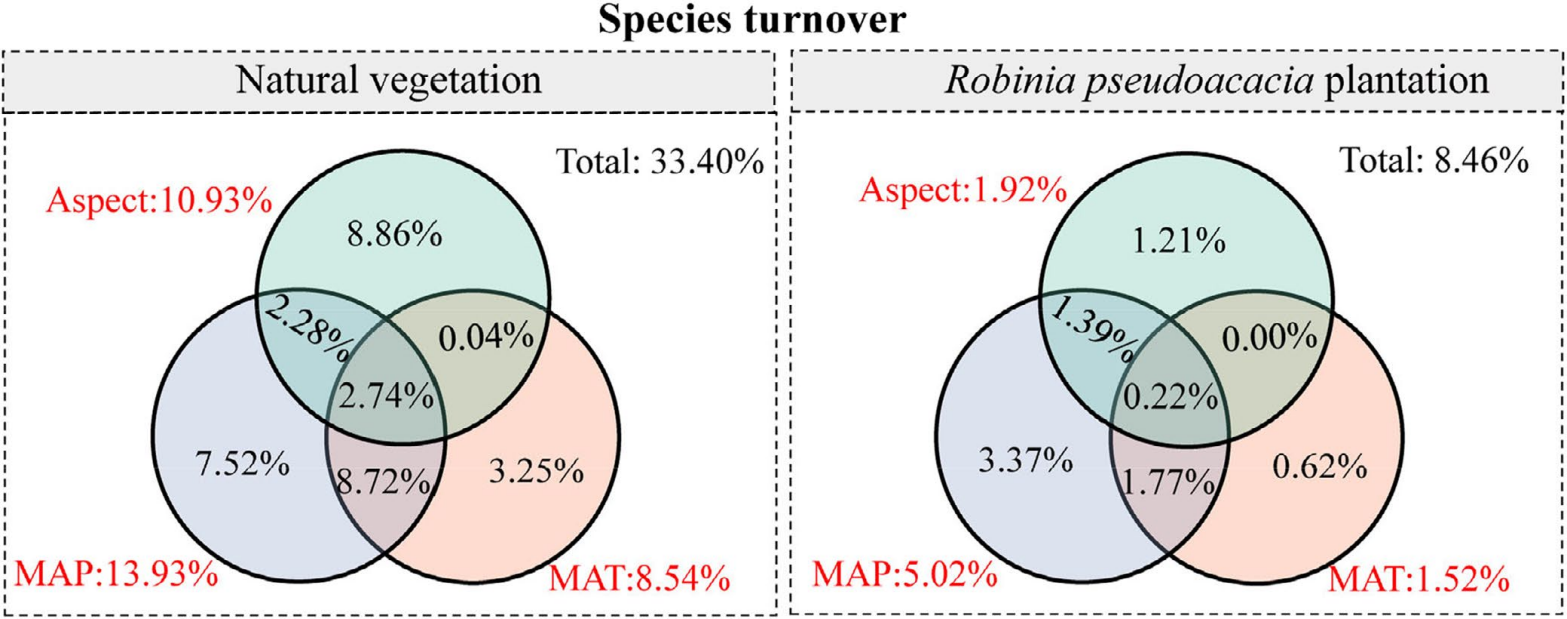
Venn diagram of variation partitioning analysis (VPA), illustrating the individual and common effects of mean annual precipitation (MAP), mean annual temperature (MAT), and aspect on species turnover in natural vegetation and *Robinia pseudoacacia* plantation.

The aggregate effect of MAP, MAT, and aspect on species turnover in natural vegetation and *R. pseudoacacia* plantation was 33.40% and 8.46%, respectively. In natural vegetation, the individual effects of MAT, MAP, and aspect on the species turnover variation were 8.54%, 13.93%, and 10.93%, respectively. In *R. pseudoacacia* plantation, the individual effects of MAT, MAP, and aspect on the species turnover variation were 1.52%, 5.02%, and 1.92%, respectively. MAP and MAT jointly explained the majority of the species turnover variation in natural vegetation, with a small common effect of 2.74% shared among the MAT, MAP, and aspect (Figure 6).

## 4 Discussion

Our findings underscore the critical importance of ITV in understanding how plant communities respond to environmental variations. This concept, increasingly recognized in ecological research (Crutsinger et al. 2006; Wang et al. 2023), complements traditional species turnover‐focused approaches by providing a nuanced perspective on community dynamics. While numerous studies emphasize species turnover as the dominant driver of community‐level functional changes (McGill et al. 2006; Puglielli et al. 2022), our results revealed that ITV can often surpass species turnover in shaping functional composition. By simultaneously quantifying both mechanisms, we advance the current understanding of plant community assembly and adaptation in the context of vegetation restoration.

Our study demonstrated that ITV explains substantial variation in both leaf and root traits, frequently exceeding the contribution of species turnover (Figure 2). This highlights the adaptive capacity of individual species to adjust their traits in response to environmental gradients. Notably, ITV in leaf chemical traits—such as nutrient content—was greater than in morphological traits. This pattern aligns with previous research suggesting that chemical traits are more plastic and responsive to resource availability than morphological traits (Lin et al. 2020; Siefert et al. 2015). Such plasticity likely reflects the ecological imperative for rapid, context‐dependent optimization of nutrient acquisition and allocation (Rozendaal et al. 2006; Siefert et al. 2015). Similarly, the pivotal role of root trait variability underscores a growing consensus that belowground traits are critical for understanding community responses to environmental variation (Bardgett et al. 2014; Kou et al. 2018). Incorporating both aboveground and belowground traits is therefore essential to fully capture the breadth of functional responses in plant communities. Neglecting ITV risks underestimating the capacity of communities to adapt to changing environments (Violle et al. 2012; Zheng et al. 2022).

The relative contributions of ITV and species turnover varied notably between natural and *R. pseudoacacia* plantation communities. *Robinia pseudoacacia* plantation, dominated by *R. pseudoacacia* , exhibited a higher relative importance of ITV compared to natural vegetation. This likely reflects the reduced species richness of artificial communities, where dominant species with broad phenotypic plasticity exert a disproportionate influence on functional composition (Pan et al. 2022; Violle et al. 2007). Such plasticity may enhance the resilience of these communities to environmental variability (Mason et al. 2012). This finding is consistent with the observation by Siefert et al. (2015) that the relative contribution of ITV decreases as species richness increases. In contrast, natural vegetation, shaped over longer timescales by processes such as environmental filtering and interspecific competition, appeared to rely more heavily on species turnover. This reliance suggests that natural communities are more sensitive to shifts in species composition, resulting in plant communities with high environmental adaptability after environmental filtering (Yang et al. 2014). These findings highlight the need for restoration strategies that account for the distinct dynamics of artificial and natural systems to optimize their resilience and functionality.

Our results also highlight complex interactions between ITV and species turnover, with covariation patterns varying across traits. Positive covariation for specific leaf traits suggests that ITV and species turnover can act synergistically to reinforce trait‐environment relationships (Kichenin et al. 2013). In these cases, environmental factors select for both phenotypically flexible species and shifts in species composition, jointly promoting trait values that enhance resource use efficiency (Zuo et al. 2017). Conversely, we observed negative covariation for certain root traits, indicating compensatory interactions between ITV and species turnover. This suggests that when one mechanism is constrained, the other may stabilize community‐level trait distributions, maintaining functional stability under fluctuating environmental conditions (Pérez‐Ramos et al. 2012). Understanding these interactions is crucial for predicting community dynamics and ecosystem functioning under global environmental change.

The Loess Plateau, characterized by ecological fragility and variable climatic conditions (Wu et al. 2020), provides an ideal case study for exploring the implications of ITV in vegetation restoration. Our findings demonstrate that incorporating ITV into restoration strategies can improve the design and implementation of restoration programs. For instance, species selection strategies that prioritize phenotypically plastic species may enhance the resilience of artificial communities to environmental variability. Similarly, integrating ITV into predictive models of community assembly can refine management approaches, promoting functional stability and ecosystem recovery (Jung et al. 2014; Violle et al. 2012). These insights can inform restoration efforts by optimizing species selection, planting configurations, and management strategies (Gann et al. 2019; Tedesco et al. 2023). Furthermore, incorporating ITV into monitoring frameworks could help identify early indicators of community adaptation, facilitating proactive and adaptive management.

Beyond the Loess Plateau, our findings underscore the importance of incorporating ITV into trait‐based ecological research across diverse ecosystems. This approach can elucidate the mechanisms underlying community assembly, ecosystem functioning, and adaptation to global change (Rixen et al. 2022). By explicitly integrating ITV, researchers can achieve a more comprehensive understanding of the processes shaping plant communities (Lajoie and Vellend 2015). Future research should investigate the generality of our findings across different ecosystems and environmental gradients. Additionally, exploring the genetic and physiological underpinnings of ITV could provide deeper insights into the mechanisms enabling species to adjust to changing environments. Such efforts are essential for advancing trait‐based ecology and informing conservation and restoration practices in a rapidly changing world.

## 5 Conclusion

Our findings clearly demonstrate that ITV is a more influential driver of community functional composition than species turnover, particularly in *R. pseudoacacia* plantation communities. While both ITV and species turnover contribute to shifts in trait values, ITV consistently accounted for a larger proportion of variation in both leaf and root functional traits. The observed positive covariation between ITV and species turnover for certain leaf traits highlights the potential for synergistic effects, where inter‐ and intraspecific processes jointly shape community responses to environmental variation. By emphasizing the predominance of ITV in determining functional composition, this study underscores the importance of incorporating ITV into trait‐based ecological frameworks. Doing so will enhance our understanding of community assembly processes and ecosystem functioning, offering valuable insights for ecological restoration strategies. These findings are particularly relevant for the Loess Plateau but have broader implications for restoration and management efforts across diverse ecosystems.

## Author Contributions

**Yuting Yang:** data curation (equal), investigation (equal), methodology (equal), writing – original draft (lead), writing – review and editing (lead). **Zhifei Chen:** formal analysis (equal), investigation (equal), methodology (equal). **Xiangtao Wang:** data curation (equal), methodology (equal). **Junqin Li:** formal analysis (equal), methodology (equal). **Yang Gao:** data curation (equal), formal analysis (supporting). **Puchang Wang:** funding acquisition (equal), writing – review and editing (equal). **Zhongming Wen:** conceptualization (equal), funding acquisition (equal), writing – review and editing (equal).

## Conflicts of Interest

The authors declare no conflicts of interest.

## Supporting information


Data S1:

## Data Availability

The data supporting the results used to generate the analyses are available at the Supporting Information S1.

## References

[ece371808-bib-0001] Araújo, M. S. , D. I. Bolnick , and C. A. Layman . 2011. “The Ecological Causes of Individual Specialisation.” Ecology Letters 14, no. 9: 948–958. 21790933 10.1111/j.1461-0248.2011.01662.x

[ece371808-bib-0002] Bardgett, R. D. , L. Mommer , and F. T. De Vries . 2014. “Going Underground: Root Traits as Drivers of Ecosystem Processes.” Trends in Ecology & Evolution 29, no. 12: 692–699. 25459399 10.1016/j.tree.2014.10.006

[ece371808-bib-0004] Crutsinger, G. M. , M. D. Collins , J. A. Fordyce , Z. Gompert , C. C. Nice , and N. J. Sanders . 2006. “Plant Genotypic Diversity Predicts Community Structure and Governs an Ecosystem Process.” Science 313: 966–968. 16917062 10.1126/science.1128326

[ece371808-bib-0005] Díaz, S. 2025. “Plant Functional Traits and the Entangled Phenotype.” Functional Ecology 00: 1–16.

[ece371808-bib-0006] Feng, X. M. , B. J. Fu , S. Piao , et al. 2016. “Revegetation in China's Loess Plateau Is Approaching Sustainable Water Resource Limits.” Nature Climate Change 6, no. 11: 1019–1022.

[ece371808-bib-0007] Fenollosa, E. , P. Fernandes , A. Hector , et al. 2024. “Differential Responses of Community‐Level Functional Traits to Mid‐ and Late‐Season Experimental Drought in a Temperate Grassland.” Journal of Ecology 112, no. 10: 2292–2306.

[ece371808-bib-0008] Fu, B. J. , Y. X. Liu , and M. E. Meadows . 2023. “Ecological Restoration for Sustainable Development in China.” National Science Review 10, no. 7: nwad033. 37266558 10.1093/nsr/nwad033 PMC10232043

[ece371808-bib-0009] Funk, J. L. , J. E. Larson , G. M. Ames , et al. 2017. “Revisiting the Holy Grail: Using Plant Functional Traits to Understand Ecological Processes.” Biological Reviews 92, no. 2: 1156–1173. 27103505 10.1111/brv.12275

[ece371808-bib-0010] Gann, G. D. , T. McDonald , B. Walder , et al. 2019. “International Principles and Standards for the Practice of Ecological Restoration.” Restoration Ecology 27: S1–S46.

[ece371808-bib-0011] Huang, L. , and M. Shao . 2019. “Advances and Perspectives on Soil Water Research in China's Loess Plateau.” Earth‐Science Reviews 199: 102962.

[ece371808-bib-0012] Hurtado, P. , M. Prieto , G. Aragón , F. de Bello , and I. Martínez . 2020. “Intraspecific Variability Drives Functional Changes in Lichen Epiphytic Communities Across Europe.” Ecology 101: e03017. 32080841 10.1002/ecy.3017

[ece371808-bib-0013] Jia, X. X. , X. Bai , C. G. Liu , C. L. Zhao , M. Shao , and Y. H. Pan . 2024. “Differences in Plant Water Use Between Check‐Dam Land and Slope Land on the Loess Plateau: Significance for Vegetation Restoration.” Agriculture, Ecosystems & Environment 362: 108849.

[ece371808-bib-0014] Jian, S. Q. , C. Y. Zhao , S. M. Fang , and K. Yu . 2015. “Effects of Different Vegetation Restoration on Soil Water Storage and Water Balance in the Chinese Loess Plateau.” Agricultural and Forest Meteorology 206: 85–96.

[ece371808-bib-0015] Jung, V. , C. H. Albert , C. Violle , G. Kunstler , G. Loucougaray , and T. Spiegelberger . 2014. “Intraspecific Trait Variability Mediates the Response of Subalpine Grassland Communities to Extreme Drought Events.” Journal of Ecology 102, no. 1: 45–53.

[ece371808-bib-0016] Kichenin, E. , D. A. Wardle , D. A. Peltzer , C. W. Morse , and G. T. Freschet . 2013. “Contrasting Effects of Plant Inter‐ and Intraspecific Variation on Community‐Level Trait Measures Along an Environmental Gradient.” Functional Ecology 27, no. 5: 1254–1261.

[ece371808-bib-0017] Kou, L. , L. Jiang , X. Fu , X. Dai , H. Wang , and S. Li . 2018. “Nitrogen Deposition Increases Root Production and Turnover but Slows Root Decomposition in *Pinus elliottii* Plantations.” New Phytologist 218, no. 4: 1450–1461. 29512162 10.1111/nph.15066

[ece371808-bib-0018] Lajoie, G. , and M. Vellend . 2015. “Understanding Context Dependence in the Contribution of Intraspecific Variation to Community Trait‐Environment Matching.” Ecology 96: 2912–2922. 27070011 10.1890/15-0156.1

[ece371808-bib-0019] Lang, B. , J. Ahlborn , M. Oyunbileg , et al. 2020. “Grazing Effects on Intraspecific Trait Variability Vary With Changing Precipitation Patterns in Mongolian Rangelands.” Ecology and Evolution 10, no. 2: 678–691. 32015835 10.1002/ece3.5895 PMC6988561

[ece371808-bib-0020] Laughlin, D. C. 2014. “Applying Trait‐Based Models to Achieve Functional Targets for Theory‐Driven Ecological Restoration.” Ecology Letters 17, no. 7: 771–784. 24766299 10.1111/ele.12288

[ece371808-bib-0021] Lepš, J. , F. de Bello , P. Šmilauer , and J. Doležal . 2011. “Community Trait Response to Environment: Disentangling Species Turnover vs Intraspecific Trait Variability Effects.” Ecography 34, no. 5: 856–863.

[ece371808-bib-0022] Lin, G. , D. Zeng , and R. Mao . 2020. “Traits and Their Plasticity Determine Responses of Plant Performance and Community Functional Property to Nitrogen Enrichment in a Boreal Peatland.” Plant and Soil 449, no. 1–2: 151–167.

[ece371808-bib-0023] Liu, H. , D. Yin , P. He , M. Cadotte , and Q. Ye . 2024. “Linking Plant Functional Traits to Biodiversity Under Environmental Change.” Biological Diversity 1: 22–28.

[ece371808-bib-0024] Lü, X. T. , Y. Y. Hu , H. Y. Zhang , et al. 2018. “Intraspecific Variation Drives Community‐Level Stoichiometric Responses to Nitrogen and Water Enrichment in a Temperate Steppe.” Plant and Soil 423: 307–315.

[ece371808-bib-0025] Mason, N. W. H. , S. J. Richardson , D. A. Peltzer , F. de Bello , D. A. Wardle , and R. B. Allen . 2012. “Changes in Coexistence Mechanisms Along a Long‐Term Soil Chronosequence Revealed by Functional Trait Diversity.” Journal of Ecology 100: 678–689.

[ece371808-bib-0026] McGill, B. J. , B. J. Enquist , E. Weiher , and M. Westoby . 2006. “Rebuilding Community Ecology From Functional Traits.” Trends in Ecology & Evolution 21, no. 4: 178–185. 16701083 10.1016/j.tree.2006.02.002

[ece371808-bib-0027] Niu, K. C. , J. S. He , and M. J. Lechowicz . 2016. “Grazing‐Induced Shifts in Community Functional Composition and Soil Nutrient Availability in Tibetan Alpine Meadows.” Journal of Applied Ecology 53, no. 5: 1554–1564.

[ece371808-bib-0028] Pan, Q. , Z. Wen , T. Wu , et al. 2022. “Trade‐Offs and Synergies of Forest Ecosystem Services From the Perspective of Plant Functional Traits: A Systematic Review.” Ecosystem Services 58: 101484.

[ece371808-bib-0029] Pérez‐Ramos, I. , C. Roumet , P. Cruz , A. Blanchard , P. Autran , and E. Garnier . 2012. “Evidence for a ‘Plant Community Economics Spectrum’ Driven by Nutrient and Water Limitations in a Mediterranean Rangeland of Southern France.” Journal of Ecology 100: 1315–1327.

[ece371808-bib-0030] Puglielli, G. , A. Bricca , S. Chelli , et al. 2024. “Intraspecific Variability of Leaf Form and Function Across Habitat Types.” Ecology Letters 27, no. 3: e14396. 38456670 10.1111/ele.14396

[ece371808-bib-0031] Puglielli, G. , C. P. Carmona , L. Varone , L. Laanisto , and C. Ricotta . 2022. “Phenotypic Dissimilarity Index: Correcting for Intra‐ and Interindividual Variability When Quantifying Phenotypic Variation.” Ecology 103: e3806. 35791858 10.1002/ecy.3806

[ece371808-bib-0032] Rixen, C. , S. Wipf , S. B. Rumpf , et al. 2022. “Intraspecific Trait Variation in Alpine Plants Relates to Their Elevational Distribution.” Journal of Ecology 110, no. 4: 860–875.

[ece371808-bib-0033] Rozendaal, D. M. A. , V. H. Hurtado , and L. Poorter . 2006. “Plasticity in Leaf Traits of 38 Tropical Tree Species in Response to Light; Relationships With Light Demand and Adult Stature.” Functional Ecology 13, no. 20: 207–216.

[ece371808-bib-0034] Sanders, T. , M. Solan , and J. A. Godbold . 2025. “Intraspecific Variability Across Seasons and Geographically Distinct Populations Can Modify Species Contributions to Ecosystems.” Functional Ecology 39, no. 3: 698–710.

[ece371808-bib-0035] Siefert, A. , C. Violle , L. Chalmandrier , et al. 2015. “A Global Meta‐Analysis of the Relative Extent of Intraspecific Trait Variation in Plant Communities.” Ecology Letters 18, no. 12: 1406–1419. 26415616 10.1111/ele.12508

[ece371808-bib-0036] Su, B. , Z. Su , and Z. Shangguan . 2021. “Trade‐Off Analyses of Plant Biomass and Soil Moisture Relations on the Loess Plateau.” Catena 197: 104946.

[ece371808-bib-0037] Tedesco, A. M. , S. López‐Cubillos , R. Chazdon , et al. 2023. “Beyond Ecology: Ecosystem Restoration as a Process for Social‐Ecological Transformation.” Trends in Ecology & Evolution 38, no. 7: 643–653. 36898928 10.1016/j.tree.2023.02.007

[ece371808-bib-0038] Violle, C. , B. J. Enquist , B. J. McGill , et al. 2012. “The Return of the Variance: Intraspecific Variability in Community Ecology.” Trends in Ecology & Evolution 27, no. 4: 244–252. 22244797 10.1016/j.tree.2011.11.014

[ece371808-bib-0039] Violle, C. , M.‐L. Navas , D. Vile , et al. 2007. “Let the Concept of Trait Be Functional.” Oikos 116, no. 5: 882–892.

[ece371808-bib-0040] Wang, C. A. , X. Li , X. M. Lu , Y. Wang , and Y. F. Bai . 2023. “Intraspecific Trait Variation Governs Grazing‐Induced Shifts in Plant Community Above‐ and Below‐Ground Functional Trait Composition.” Agriculture, Ecosystems & Environment 346: 108357.

[ece371808-bib-0041] Wang, X. , J. Zhang , X. Yan , et al. 2022. “Nitrogen Enrichment and Warming Shift Community Functional Composition via Distinct Mechanisms: The Role of Intraspecific Trait Variability and Species Turnover.” Functional Ecology 36, no. 5: 1230–1242.

[ece371808-bib-0042] Wu, X. T. , Y. P. Wei , B. J. Fu , S. Wang , Y. Zhao , and E. F. Moran . 2020. “Evolution and Effects of the Social‐Ecological System Over a Millennium in China's Loess Plateau.” Science Advances 6, no. 41: eabc0276. 33028526 10.1126/sciadv.abc0276 PMC7541069

[ece371808-bib-0043] Yan, P. , N. P. He , K. L. Yu , L. Xu , and K. Van Meerbeek . 2023. “Integrating Multiple Plant Functional Traits to Predict Ecosystem Productivity.” Communications Biology 6, no. 1: 239. 36869238 10.1038/s42003-023-04626-3 PMC9984401

[ece371808-bib-0044] Yang, L. , W. Wei , L. Chen , W. Chen , and J. Wang . 2014. “Response of Temporal Variation of Soil Moisture to Vegetation Restoration in Semi‐Arid Loess Plateau, China.” Catena 115: 123–133.

[ece371808-bib-0045] Yang, Y. , Y. Fan , C. M. Basang , J. Lu , C. Zheng , and Z. Wen . 2022. “Different Biomass Production and Soil Water Patterns Between Natural and Artificial Vegetation Along an Environmental Gradient on the Loess Plateau.” Science of the Total Environment 814: 152839. 34995600 10.1016/j.scitotenv.2021.152839

[ece371808-bib-0046] Zheng, S. , Y. Chi , X. Yang , W. Li , Z. Lan , and Y. Bai . 2022. “Direct and Indirect Effects of Nitrogen Enrichment and Grazing on Grassland Productivity Through Intraspecific Trait Variability.” Journal of Applied Ecology 59, no. 2: 598–610.

[ece371808-bib-0047] Zuo, X. , X. Yue , P. Lv , et al. 2017. “Contrasting Effects of Plant Inter‐ and Intraspecific Variation on Community Trait Responses to Restoration of a Sandy Grassland Ecosystem.” Ecology and Evolution 7: 1125–1134. 28303183 10.1002/ece3.2711 PMC5306005

