# Supplementary material for: Contrasting Roles of Intraspecific Trait Variability and Species Turnover in Shaping Functional Composition During Vegetation Restoration on the Loess Plateau
Source: Ecol Evol. 2025 Jul 13;15(7):e71808. doi: 10.1002/ece3.71808 (PMC12256144; doi:10.1002/ece3.71808)
Supplement: Supplementary file 1 — Data S1: [file ECE3-15-e71808-s001.zip › Supporting Information for review and publication.docx]

Table S1 The dominant species in the Yanhe River catchment

| Growth form | Species latin name |
| --- | --- |
| Herb | *Stipa grandis; Lespedeza bicolor; Bothriochloa ischaemum; Artemisia capillaris; Stipa bungeana; Artemisia mongolica; Artemisia scoparia; Aster altaicus; Poa annua; Potentilla tanacetifolia; Leymus secalinus; Pennisetum flaccidum; Artemisia gmelinii; Pulsatilla chinensis; Thymus mongolicus; Cleistogenes hancei; Astragalus scaberrimus; Cleistogenes squarrosa; Astragalus melilotoides; Cirsium arvense var. integrifolium; Cleistogenes caespitosa; Lespedeza floribunda; Elymus kamoji; Oxytropis bicolor; Potentilla discolor; Calamagrostis epigejos; Glycyrrhiza uralensis; Setaria viridis; Polygonatum sibiricum; Astragalus discolor; Leontopodium leontopodioides; Neotrinia splendens; Lysimachia barystachys; Geranium wilfordii; Artemisia frigida; Solanum nigrum; Phragmites australis; Klasea centauroides; Carex lancifolia; Rubia cordifolia; Adenophora stricta; Oxytropis racemosa; Potentilla chinensis; Bupleurum hamiltonii; Chrysanthemum chanetii; Lespedeza davurica; Linum usitatissimum; Fragaria vesca; Allium chrysanthum; Arundinella hirta; Vicia sepium; Patrinia heterophylla; Leonurus japonicus; Siphonostegia chinensis; Poa sphondylodes; Iris tectorum; Polygala tenuifolia; Viola dactyloides; Stipa capillata; Selaginella sinensis; Salsola collina; Viola philippica;* |
| Shrub | *Vitex negundo var. heterophylla; Sophora davidii; Prinsepia utilis; Melilotus suaveolens; Periploca sepium; Clematis fruticosa; Ostryopsis davidiana; Buddleja alternifolia; Rosa xanthina; Viburnum dilatatum; Caragana sinica; Rhamnus erythroxylum; Zabelia biflora; Elaeagnus umbellata; Wikstroemia canescens; Lonicera japonica; Hippophae rhamnoides; Ampelopsis glandulosa; Cotoneaster multiflorus; Ziziphus jujuba var. spinosa; Spiraea ouensanensis; Rosa rubus; Leptodermis potaninii; Crataegus cuneata;* |
| Tree | *Platycladus orientalis; Ailanthus altissima; Robinia pseudoacacia; Pyrus betulifolia; Acer palmatum; Quercus mongolica; Acer buergerianum; Prunus davidiana; Populus avidiana; Xanthoceras sorbifolium; Prunus pseudocerasus; Ulmus pumila;* |

Table S2 The range and variation of community weighted mean traits for natural vegetation and *Robinia pseudoacacia* plantation

| Functional traits | Natural vegetation | | *Robinia pseudoacacia* plantation | |
| --- | --- | --- | --- | --- |
|  | mean ± s.e. | range | mean ± s.e. | range |
| SLA (cm^2^ g^-1^) | 123.22±2.43 | 46.58~214.76 | 173.92±7.22 | 53.76~276.44 |
| LTD (g cm^-3^) | 0.57±0.02 | 0.06~1.65 | 0.42±0.03 | 0.11~1.14 |
| SRL (m g^-1^) | 6.94±0.61 | 2.27~16.82 | 5.01±0.60 | 2.28~9.76 |
| RTD (g cm^-3^) | 0.40±0.02 | 0.06~0.77 | 0.36±0.02 | 0.21~0.50 |
| LN (g kg^-1^) | 19.20±0.40 | 6.55~37.25 | 26.56±1.01 | 11.31~37.80 |
| LP (g kg^-1^) | 1.30±0.04 | 0.48~4.05 | 2.15±0.15 | 0.89~5.39 |
| LN:P | 16.10±0.34 | 3.37~32.88 | 14.45±0.79 | 5.67~27.27 |
| RN (g kg^-1^) | 8.08±0.22 | 3.81~18.37 | 10.28±0.89 | 5.96~17.49 |
| RP (g kg^-1^) | 0.75±0.04 | 0.31~2.28 | 1.21±0.21 | 0.45~3.43 |
| RN:P | 12.14±0.54 | 2.44~31.82 | 12.13±2.12 | 2.79~32.22 |

SLA, specific leaf area; LTD, leaf tissue density; LN, leaf nitrogen content; LP, leaf phosphorus content; LN:P, leaf N:P ratio. SRL, specific root length; RTD, root tissue density; RN, root nitrogen content; RP, root phosphorus content; RN:P, root N:P

Figure S1 The variation in environmental variables in the Yanhe River catchment


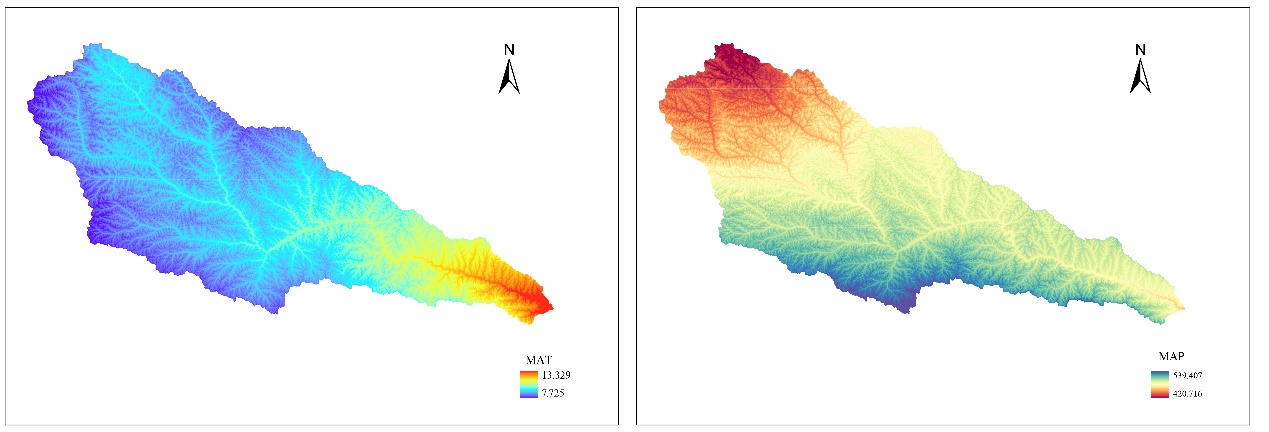


Figure S2 The functional traits change with environmental factors


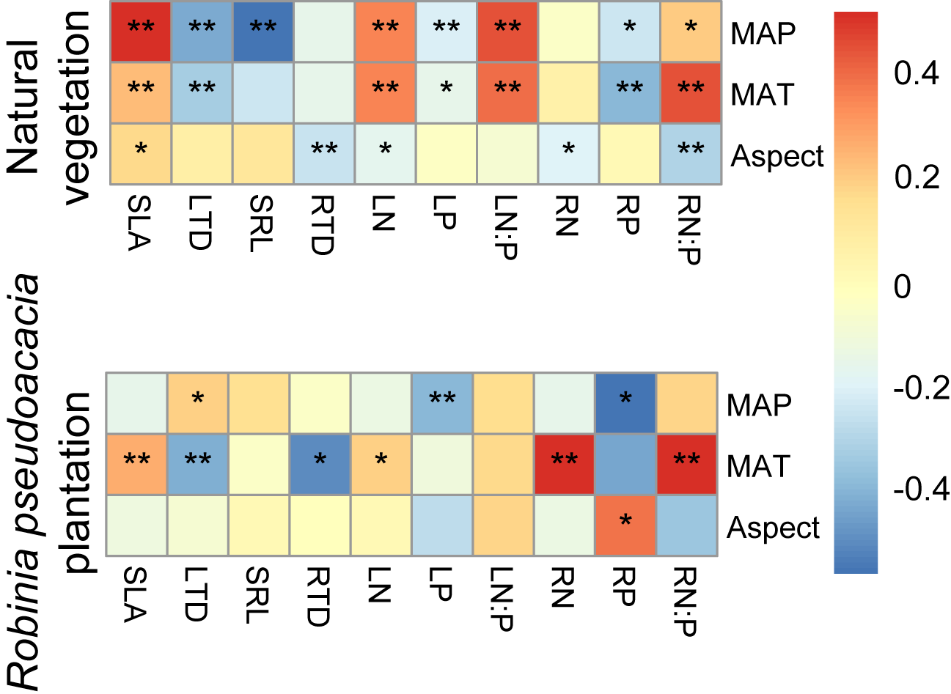


MAP, mean annual precipitation; MAT, mean annual temperature; SLA, specific leaf area; LTD, leaf tissue density; LN, leaf nitrogen content; LP, leaf phosphorus content; LN:P, leaf N:P ratio. SRL, specific root length; RTD, root tissue density; RN, root nitrogen content; RP, root phosphorus content; RN:P, root N:P
